# Pathway enrichment in genome‐wide analysis of longitudinal Alzheimer's disease biomarker endophenotypes

**DOI:** 10.1002/alz.14308

**Published:** 2024-10-23

**Authors:** Thea J. Rosewood, Kwangsik Nho, Shannon L. Risacher, Shiwei Liu, Sujuan Gao, Li Shen, Tatiana Foroud, Andrew J. Saykin, for the Alzheimer's Disease Neuroimaging Initiative

**Affiliations:** ^1^ Indiana Alzheimer's Disease Research Center Indianapolis Indiana USA; ^2^ Department of Medical and Molecular Genetics Indiana University School of Medicine Indianapolis Indiana USA; ^3^ Department of Radiology and Imaging Sciences Indiana University School of Medicine Indianapolis Indiana USA; ^4^ School of Informatics and Computing Indiana University Indianapolis Indiana USA; ^5^ Department of Biostatistics Indiana University School of Medicine Indianapolis Indiana USA; ^6^ Department of Biostatistics Epidemiology and Informatics Perelman School of Medicine Philadelphia Pennsylvania USA

**Keywords:** endophenotypes, genetics, genome‐wide association study, longitudinal, pathway

## Abstract

**INTRODUCTION:**

The genetic pathways that influence longitudinal heterogeneous changes in Alzheimer's disease (AD) may provide insight into disease mechanisms and potential therapeutic targets.

**METHODS:**

Longitudinal endophenotypes from the Alzheimer's Disease Neuroimaging Initiative (ADNI) representing amyloid, tau, neurodegeneration (A/T/N), and cognition were selected. Genome‐wide association analysis was performed using a linear mixed model (LMM) approach, followed by gene and pathway enrichment with significant and functionally relevant SNPs.

**RESULTS:**

A total of 33 and 19 statistically significant pathways were identified associating with the intercept and longitudinal trajectory, respectively. The longitudinal intercept pathways represent eight groups: immune, metabolic, cell growth and survival, DNA maintenance, neuronal signaling, *R* *AS*/*MAPK*/*ERK signaling pathways*, vesicle and lysosomal transport, and transcription modification. Longitudinal trajectory pathways represented six groups: Immune, metabolic, cell signaling, cytoskeleton, and glycosylation.

**DISCUSSION:**

Longitudinal enrichment identified pathways that uniquely associate with trajectories of key AD biomarkers and cognition, providing new insight into AD course‐related mechanisms and potential new therapeutic targets.

**Highlights:**

- A systematic genome‐wide analysis with longitudinal AD biomarker endophenotypes was performed.
- Enriched pathways were identified with functionally derived SNP to gene analysis.
- Fifty‐two pathways were associated with longitudinal trajectory and intercept.
- Many of the identified pathways are specific steps in larger pathways implicated in AD.
- The identified pathways may provide therapeutic targets and areas for further study.

## 1 BACKGROUND

The heterogeneous nature and course of Alzheimer's disease (AD) have been a barrier to a fundamental understanding of the disease, especially regarding the role of genetic variation. Studies have shown that disease trajectory, the rate and severity of disease progression, varies across individuals.^1^, ^2^ The heterogeneity in longitudinal trajectory is influenced by a variety of factors, and studies are needed to examine the role of genetic variation in disease course.^3^

Previously, we applied an endophenotype approach to cross‐sectional data to identify genetic markers associated with key AD endophenotypes from the Alzheimer's Disease Neuroimaging Initiative (ADNI).^4^ While the cross‐sectional single nucleotide polymorphism (SNP) by a diagnosis interaction approach in that study touched on some of the changes that can happen over the course of disease progression, a cross‐sectional interaction could not evaluate the full longitudinal trajectory of those endophenotypes. The ADNI cohort has gathered a large amount of longitudinal data across participants, providing an opportunity to evaluate the genetic effects on longitudinal trajectory. Following the National Institute on Aging‐Alzheimer's Association (NIA‐AA) research framework, we evaluated biomarker endophenotypes that fall under the following groups: amyloid beta (Aβ) deposition (A), pathologic or phosphorylated tau (T), neurodegeneration (N), and cognition (C).^5^

Longitudinal analysis introduces new challenges to performing comprehensive genetic studies. There are various ways to interrogate longitudinal data, with different models offering different strengths and limitations. In this study, we utilized a linear mixed model (LMM) approach to evaluate the longitudinal trajectory across the ADNI cohort, representing different stages of the disease. This approach enabled us to assess endophenotypes over time, with varying observation points among participants. With this, we evaluated the interaction between genetic effects and time. The SNP by Time (SNP×Time) interaction provided insights into genetic influences on endophenotype trajectories over the observed period.

To further interpret these data, we employed gene enrichment and pathway enrichment approaches. The SNP‐to‐gene assignment method we used enhances the signal of genetic markers within genes and those with known expression effects in brain tissue. This approach allowed us to identify genes and pathways with strong evidence for functional relevance in AD.

In this study, we aimed to identify genetic effects that influence AD trajectory. The genetic markers and pathways that we identified may provide insights into how disease progression is affected and potentially provide therapeutic targets to help mitigate AD pathology.

## 2 METHODS

### 2.1 Study participants

Participants from the ADNI Phase 1 (ADNI‐1) and its subsequent extensions (ADNI‐GO/2/3)^6^, ^7^ were included in this study. Further information about these studies, participant enrollment, protocols, and other material can be found at the ADNI website.^8^ Written informed consent was obtained from all participants, and all protocols were approved by each participating study and the site's Institutional Review Board.

### 2.2 Genotyping and imputation

The ADNI participants were genotyped on the Illumina Human 610‐Quad BeadChip, the Illumina HumanOmniExpress Beadchip, the Illumina Omni 2.5 M platform, or the Illumina Global Screening Array BeadChip (Illumina, Inc., San Diego, CA, USA).^9^ After sample and SNP standard quality control procedures of genome‐wide association study (GWAS) data, genotype imputation was performed over each data set separately using the Haplotype Reference Consortium Panel r1.1. To avoid population stratification confounding, non‐Hispanic European ancestry ADNI participants (*N* = 1869) were selected for this analysis by genetic clustering using HapMap 3 genotype data and multidimensional scaling (MDS) analysis.

**RESEARCH IN CONTEXT**

1. **Systematic review**: The authors reviewed traditional literature sources, meeting presentations, and abstracts. While there are longitudinal studies of AD phenotypes, this study represents a comprehensive genome‐wide association analysis of major AD longitudinal endophenotype biomarkers.
2. **Interpretation**: Our findings identify specific pathways associated with longitudinal changes of AD endophenotypes that are relevant to broader biological processes previously implicated in AD. These specific pathways provide insight into disease mechanisms and potential therapeutic targets.
3. **Future directions**: Larger and independent samples, better temporal sampling, and alternative modeling approaches would expand knowledge of mechanisms related to progression. Improved models for temporal alignment of longitudinal data in an unbiased and biologically meaningful way would permit a better representation of trajectory related to disease onset and contributory genetic variation. Better clustering methods for identifying subtypes of longitudinal trajectory would be valuable, and model systems would help with validation and prioritization for clinical application.

### 2.3 Selected phenotypes

Biomarkers were selected based on previous studies for their association with AD pathology^10^ as well as data quality for low visit‐to‐visit variability. Longitudinal measures of 11 phenotypes were selected to represent the key markers of AD represented by the NIA‐AA research framework biomarkers A/T/N as well as C: Aβ (A), tau (T), neurodegeneration (N),^5^, ^11^ and cognition (C) categories. Aβ (A) measures are represented by one meta region of interest (ROI) standardized uptake value ratio (SUVR) for [18F]Florbetapir amyloid positron emission tomography (PET) and cerebrospinal fluid (CSF) Aβ 1‐42 peptide (Aβ1‐42), tau (T) measures by CSF total tau (t‐tau) and phosphorylated tau (p‐tau181), neurodegeneration (N) by magnetic resonance imaging (MRI) atrophy measures (four ROIs) and fluorodeoxyglucose (FDG) PET meta ROI SUVR, and cognition (C) by composite scores^12^ for memory (MEM) and executive functioning (EF). MRI ROIs were selected based on previous studies of AD pathology and progression to represent the disease across pathological stages.^13^, ^14^ Table 1 presents the full list of phenotypes and sample sizes. MRI cortical thickness measures were obtained using FreeSurfer 6 for processing ADNI MRI data. An amyloid‐PET SUVR meta‐ROI was intensity normalized to the whole cerebellum and calculated. The mean SUVR within a cortical summary region consisted of the frontal, anterior/posterior cingulate, lateral parietal, and lateral temporal regions. The FDG PET meta‐ROI was a composite of the left and right angular gyrus, bilateral posterior cingulate, and left and right inferior temporal gyrus normalized to the top 50% of the pons and cerebellar vermis reference regions.

**TABLE 1:** Selected longitudinal phenotypes.

		Genetic PC1	Genetic PC2	Sex	Education	Intracranial volume	MRI field strength^a^
Selected endophenotype	*N*						
Memory composite score	1869	•	•	•	•		
Executive function composite score	1863	•	•	•	•		
Bilateral mean frontal lobe thickness	1543	•	•	•	•	•	•
Bilateral mean parietal lobe thickness	1543	•	•	•	•	•	•
Bilateral mean medial temporal lobe thickness	1536	•	•	•	•	•	•
Bilateral mean lateral temporal lobe thickness	1536	•	•	•	•	•	•
FDG PET meta ROI SUVR	782	•	•	•			
[18F] Florbetapir amyloid PET meta ROI SUVR	772	•	•	•			
CSF amyloid‐β 1‐42 peptide	777	•	•	•			
CSF total tau	776	•	•	•			
CSF phosphorylated tau	773	•	•	•			

*Note*: List of selected endophenotypes, their *N*, and covariates applied prior to analysis indicated by •.

Abbreviations: CSF, cerebrospinal fluid; FDG, fludeoxyglucose; PET, positron emission tomography; ROI, region of interest; SUVR, standardized uptake value ratio.

^a^ Values were pre‐adjusted for MRI‐field strength where applicable.

### 2.4 Genetic association analysis with linear mixed modeling

Genetic association with longitudinal endophenotypes was performed separately for each phenotype using linear mixed modeling (LME4 R package)^15^ across 6,149,175 genotyped and imputed SNPs. The model included random intercepts and random slopes for time, with participant age at the time of observation for each data point used as the time variable. Age at observation was also included as a fixed effect to control for the overall effect of age across the dataset. SNP and SNP interaction with age at observation (SNP× Time) terms were included as fixed effects to identify the SNP's association with trajectory intercept and its interaction effect with longitudinal trajectory.

To reduce computation time, each endophenotype was pre‐adjusted for selected covariates (Table 1), sex, and the first two principal components of the genetic population to account for population genetic subtypes. Cognitive and MRI measures were additionally adjusted for education. MRI measures were adjusted for intracranial volume and MRI field strength. In our previous cross‐sectional study,^4^ MRI field strength measures were confounded with the ADNI phase. Therefore, in this study, the MRI field strength beta coefficient was calculated using only cognitively normal (CN) individuals and then applied across all MRI measures for each ROI. Pre‐adjustment was performed using a LMM without any SNP variants included. The residuals of the model combined with age and intercept effects were used as the pre‐adjusted phenotype measures.

Principal component analysis of the 11 selected phenotypes identified six principal components, explaining 85% of the variance across phenotypes. An adjusted study‐wide significance threshold was set at *p* ≤ 8.33 × 10^−9^ based on these six components. Additional consideration was given to the conventional genome‐wide threshold of *p* ≤ 5 × 10^−8^ and a suggestive association threshold of *p* ≤1 × 10^−5^ for the purpose of comparison across phenotypes and for additional analyses. For interpretation purposes, linkage disequilibrium (LD) trimming was performed to identify independent peak SNPs for a genetic region, with an *R* ^2^ value of 0.15 to identify SNPs in LD with the peak SNPs. Variants were annotated for variant positions and nearest genes using ANNOVAR (version 2020‐06‐07). Additional functional evaluation was performed using the MetaBrain expression quantitative trait loci (eQTL) project,^16^ the Genotype‐Tissue Expression (GTEx) portal, and RegulomeDB^17^ to identify potential regulatory activity.

### 2.5 Gene enrichment and pathway analysis

All SNP *p* values from the LMM were analyzed for gene enrichment using MAGMA (version 1.10).^18^ To identify gene enrichment with primarily functional outcomes, SNP‐to‐gene assignment was performed in two steps with protein‐coding genes: (1) SNPs located within a gene's boundary and (2) significant SNP eQTL for the respective gene from the MetaBrain eQTL project.^16^ The gene boundary was set as the start and end point of the gene transcription from the Ensembl genome database^19^ for genome build GRCh 37. Significant SNPs for each gene from the MetaBrain eQTL project were then added to the appropriate gene on the SNP‐to‐gene list for processing in MAGMA. The “Multi” model of MAGMA was used, combining two separate models for identifying significant genes in an aggregate *p* value: tests for top SNP associations and tests for mean SNP associations. These two models allow for the selection of genes by singular strong signals (test for the top SNP) or enrichment of multiple smaller signals associated with a gene (mean SNP associations).

Results from the gene enrichment analysis were then processed through pathway‐level analysis with the C2 curated gene set collection from the Gene Set Enrichment Analysis (GSEA) Molecular Signatures Database (MSigDB).^20^, ^21^ Significant pathways were identified using the *posthoc_qc.R* script provided by the MAGMA team, based on an alpha (significance threshold) of 0.05 after gene‐based multiple testing correction.^22^ The gene enrichment and gene set enrichment processes enable the identification of genes and their associated pathways that are significantly enriched by results in this study. These genes are functionally related to eQTLs or located within protein‐coding regions.

## 3 RESULTS

### 3.1 Genome‐wide association analysis of longitudinal endophenotypes using LMMs

**FIGURE 1:**
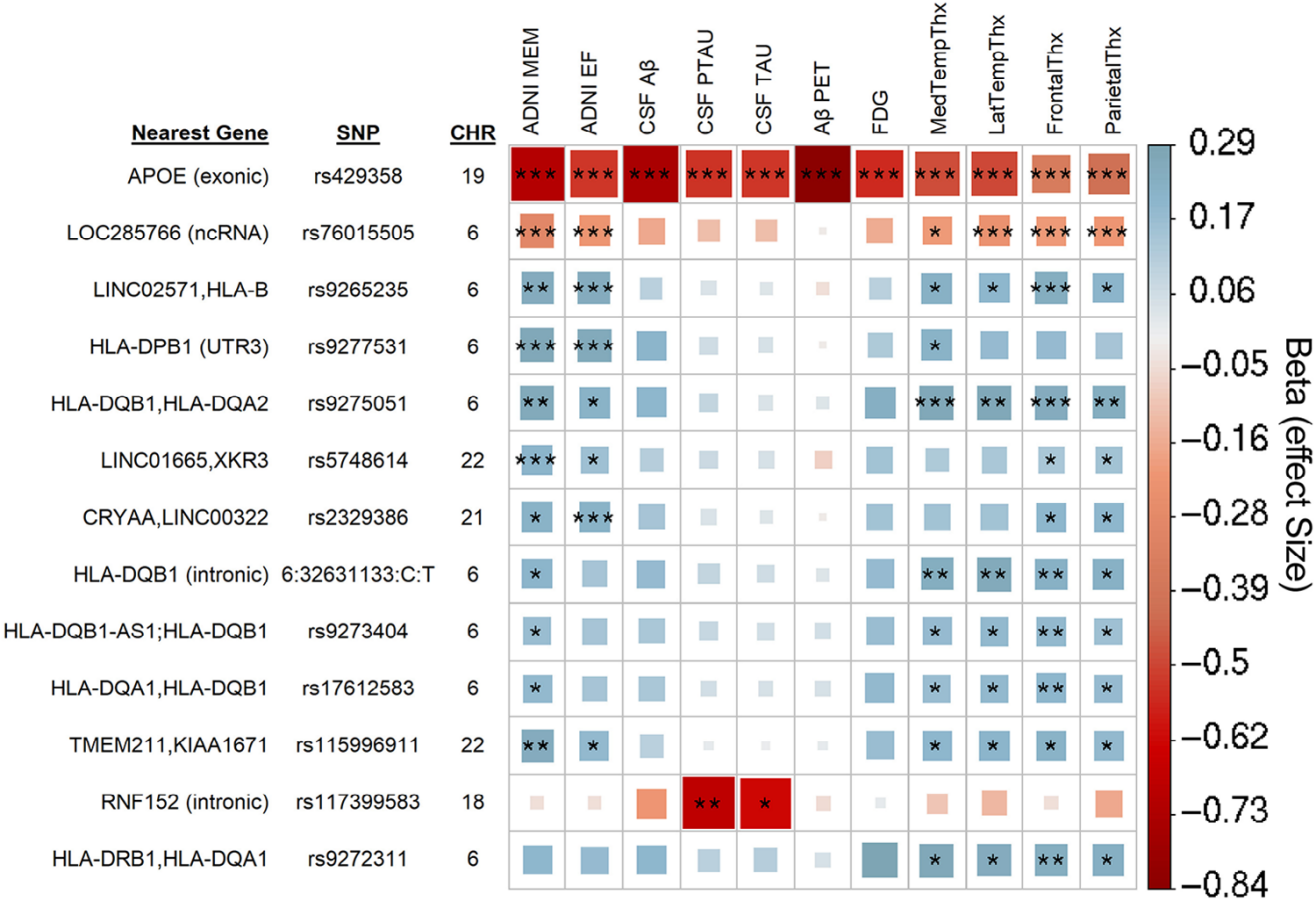
Intercept SNP effect results. Matrix of intercept results of SNP‐based analysis that met study‐wide and genome‐wide significance thresholds, trimmed to the peak SNP of each genetic region. *** indicates study‐wide significance (*p *≤ 8.33 × 10^−9^), ** conventional genome‐wide threshold (*p *≤ 5 × 10^−8^), and * suggestive association threshold (*p *≤ 1 × 10^−5^). The color and box size relate to the beta value effect size for a given association, in either a positive (blue, suggesting neuroprotective) or negative (red, suggesting neuropathological effect) direction. The two nearest genes indicate intergenic SNPs; for SNPs within gene boundaries, their location within the gene (intronic, exonic, 3′ untranslated region) is indicated in parentheses.

In the intercept results (Figure 1), a total of seven peak SNPs were identified that met study‐wide significance thresholds (*p* ≤ 8.33 × 10^−9^) after LD trimming. An additional six peak SNPs met the more conventional genome‐wide threshold (*p* ≤ 5 × 10^−8^), and 89 peak SNPs met the suggestive association threshold in at least two evaluated endophenotypes.

This study includes data from ADNI‐3, which was not present in the previous cross‐sectional analysis.^4^ Of the 27 study‐wide significant cross‐sectional SNPs, six (apolipoprotein E (*APOE)* 𝜀4 allele, rs5748614, rs76015505, rs9265235, rs9277531, rs9275051) were replicated as study‐wide significant, and 11 showed suggestive associations. Four SNPs fell just below the suggestive association threshold but still showed some significance. Differences in genotyping technology between previous ADNI stages and ADNI‐3 included in this study affected the imputation confidence for some SNPs in the new data set, accounting for the remaining top SNPs from the cross‐sectional study. Additionally, two new SNPs in this study were identified as having study‐wide significance, one primarily associated with MRI measures within or near *HLA‐DQB1* and one intergenic near *CRYAA* associated with cognitive measures, with some significance in MRI measures.

**FIGURE 2:**
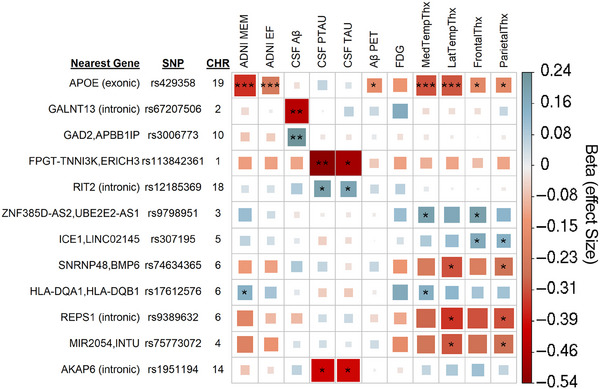
SNP×Time interaction results. Matrix of SNP×Time interaction results of SNP‐based analysis that met conventional genome‐wide association measures in at least one endophenotype or suggestive association threshold in three or more endophenotypes. *** indicates study‐wide significance (*p *≤ 8.33 × 10^−9^), ** conventional genome‐wide threshold (*p *≤ 5 × 10^−8^), and * suggestive association threshold (*p *≤ 1 × 10^−5^). The color and box size relate to the beta value effect size for a given association, in either a positive (blue, suggesting neuroprotective) or negative (red, suggesting neuropathological effect) direction. The two nearest genes indicate intergenic SNPs; for SNPs within gene boundaries, their location within the gene (intronic, exonic, 3′ untranslated region) is indicated in parentheses.

In the SNP×Time results (Figure 2), only the *APOE* allele met study‐wide significance (*p* ≤ 8.33 × 10^−9^) after LD trimming. Three additional peak SNPs met the more conventional genome‐wide threshold (*p* ≤ 5 × 10^−8^), and eight peak SNPs met the suggestive association threshold in at least two evaluated endophenotypes. Two of the genome‐wide SNPs, one within *GALNT13* and the other near *APBB1IP*, were primarily associated with CSF Aβ, while the SNP near *ERICH3* was associated with CSF tau measures.

### 3.2 Gene‐based association and gene set enrichment

**FIGURE 3:**
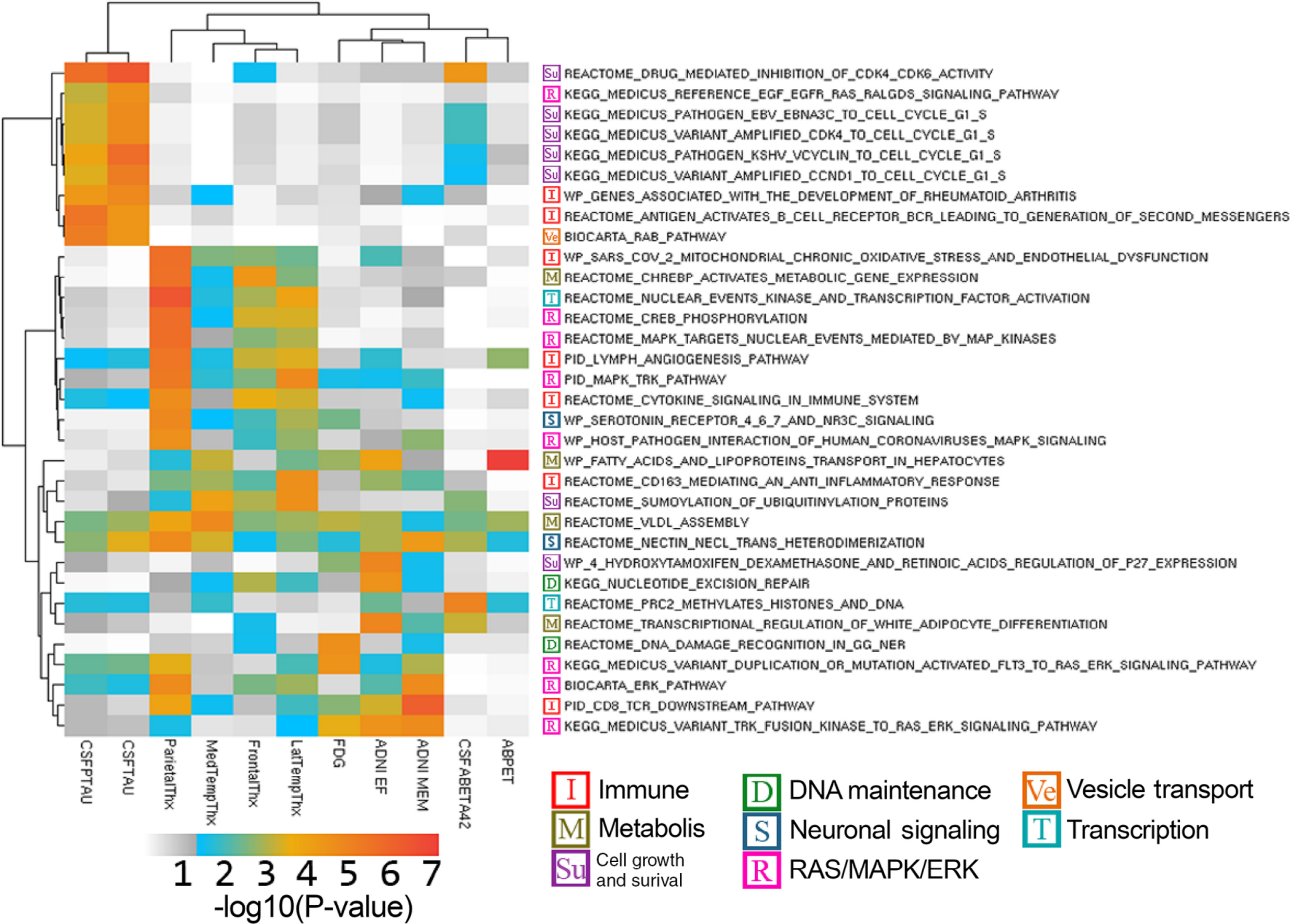
Enriched gene sets with intercept results. Heatmap of enriched gene set analysis results of intercept results. The color scale represents the log‐transformed *p* value, with white to gray representing values above the .05 threshold and blue to yellow to red indicating *p* values below the .05 threshold. WARD.D2 hierarchical clustering was applied to group together results for easier interpretation of shared patterns of association across tested endophenotypes.

**FIGURE 4:**
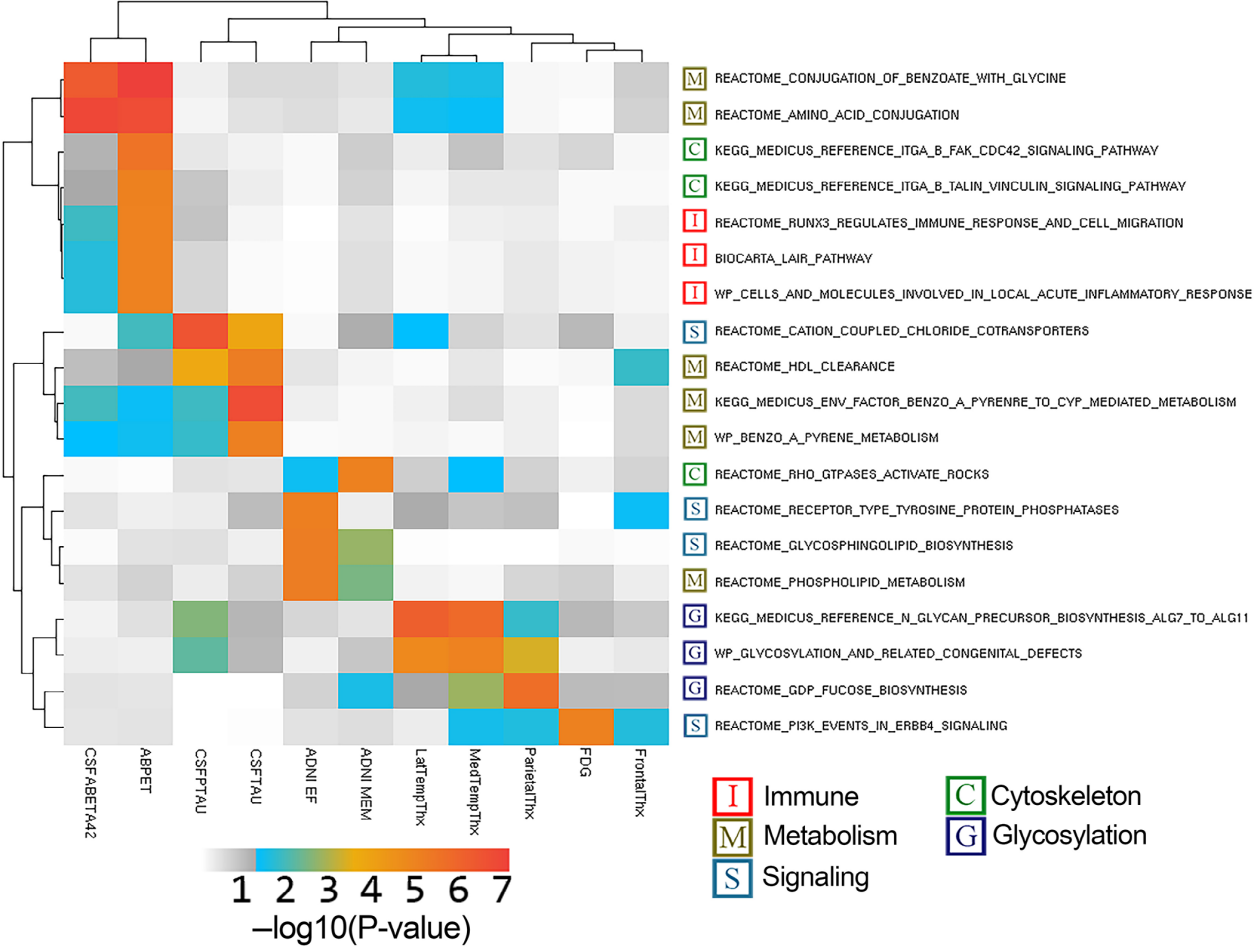
Enriched gene sets with SNP×Time results. Heatmap of enriched gene set analysis results of SNP×Time results. The color scale represents the log‐transformed *p* value, with white to gray representing values above the .05 threshold and blue to yellow to red indicating *p* values below the .05 threshold. WARD.D2 hierarchical clustering was applied to group together results for easier interpretation of shared patterns of association across tested endophenotypes.

MAGMA gene enrichment with functionally relevant SNPs identified 61 genes after false discovery rate (FDR) adjustment with the intercept term and 13 in the SNP×Time interaction term. These results were used in MAGMA gene set analysis to identify enriched genetic pathways. After MAGMA QC processes, 33 pathways were identified in the intercept term (Figure 3) and 19 in the SNP×Time interaction term (Figure 4).

Significant intercept pathways can be categorized into eight groups based on their role in larger biological processes: immune, metabolic, cell growth and survival, DNA maintenance, neuronal signaling, *RAS/MAPK/ERK* pathways, vesicle and lysosomal transport, and transcription modification. Immune pathways showed a strong effect across all measures apart from amyloid. Outside of enriched pathways driven by the *APOE* signal, metabolic pathways are associated with MRI, cognitive, and amyloid endophenotypes. Cell growth and survival pathways are largely associated with measures of tau. DNA maintenance pathways are associated with cognitive and neurodegeneration (FDG and MRI) endophenotypes. Neuronal signaling and transcription modification pathways are associated with MRI measures. One pathway related to vesicle transport, in the *RAB* pathway, is associated with tau measures. The *RAS/MAPK/ERK* signaling pathways are associated with different pathways across all measures, with one cluster largely associated with MRI measures and another with cognition and neurodegeneration.

Significant SNP×Time pathways can be categorized into five groups: immune, metabolic, cell signaling, cytoskeleton, and glycosylation. Immune and cytoskeletal pathways are largely associated with changes in amyloid trajectory. Metabolic pathways are strongly associated with changes in amyloid and tau trajectories. Glycosylation pathways are associated with changes in brain atrophy. Pathways related to cell and neuronal signaling are associated with changes in cognition, tau, and amyloid trajectories. Cell growth and survival are associated with changes in neurodegeneration trajectory.

## 4 DISCUSSION

Longitudinal association analysis of key AD endophenotypes through linear mixed modeling in this study identified novel genetic variant associations that may provide insight into genetic effects that alter AD trajectory. Those genetic variants were further analyzed to identify enriched gene sets that represent specific pathways that may be affected in disease progression.

The participants in the ADNI cohort are within the age range of AD clinical occurrence. However, many changes in AD occur before clinical symptoms manifest.^23^ This is most evident in the *APOE* 𝜀4 allele and its association with amyloid and tau measures that typically occur earlier in AD progression, with strong significance in intercept results but low significance in longitudinal trajectories for those measures. The intercept term, then, is the genetic association with the intercept of endophenotype trajectories and primarily represents changes that occurred before the observed time points. The SNP×Time genetic effects are loci that significantly alter trajectory within the observed time point.

The intercept results are similar to our previous cross‐sectional studies^4^ but may offer some new insights. The sample size in this study differs due to the longitudinal data and the inclusion of ADNI‐3, resulting in differences in significance levels. However, this analysis identified a similar association pattern and two additional significant SNPs. While tau measures did not pass any significance thresholds in the cross‐sectional analysis, a SNP intronic to *RNF152* was identified as having genome‐wide significance in this longitudinal analysis. The RING finger (RNF) family of proteins plays a role in the mTOR signaling pathway,^24^ which has been implicated in tau progression and AD.^25^, ^26^

The SNP×Time interaction results identified three genome‐wide significant SNPs within CSF measures. Two are primarily associated with CSF Aβ with poor association patterns across the remaining phenotypes, including Aβ PET data. As such, there is less confidence in their biological significance. One SNP is intronic to *GALNT13*, an O‐glycosylation gene that has shown some evidence of association with AD in epigenetic module analysis^27^ and does play a broader role in neuronal development.^28^ The final SNP is associated with CSF tau, with some signal across other endophenotypes. This SNP shows strong regulatory evidence of transcription factor binding in RegulomeDB and is a significant eQTL for *LRRIQ3* in fibroblast cell lines. HLA Class II region, including *HLA‐DQA1* and *HLA‐DQB1*, has been heavily implicated in AD.^29^, ^30^, ^31^, ^32^ This region has been particularly robust across the cross‐sectional analysis, longitudinal intercept, and suggestive association in SNP×Time analysis.

Gene enrichment and subsequent gene set enrichment provide a means to identify genetic signals that might be significant independently but represent a pattern of genetic variation functionally related to genes and gene sets. Some pathways in both intercept and SNP×Time interaction show more robust patterns of association across multiple endophenotypes. Immune pathways are strongly associated with both intercept and SNP×Time results. The immune‐related pathways are largely related to inflammation, which has long been implicated in AD,^33^, ^34^, ^35^ with findings here potentially providing insight into specific pathways of inflammation in AD brains. Metabolic pathways as well are implicated in both intercept and SNP×Time and have been strongly implicated in AD.^36^ *APOE* plays a role in metabolic pathways and is represented in some of the robust pathways in this analysis.^37^, ^38^ Other non‐*APOE*‐related pathways were identified. In the intercept, “white adipocyte differentiation” shows cross‐endophenotype association. White adipose has been shown to influence brain structure and atrophy,^39^, ^40^ play a role in inflammation in AD,^39^, ^41^ and impact vascular damage in AD.^42^, ^43^ In the SNP×Time results, “conjugation of benzoate with glycine” shows an association with amyloid and MRI measures. Benzoic acid and sodium benzoate are common preservatives, and this pathway is involved in detoxification.^44^, ^45^ Sodium benzoate has been shown to have a positive therapeutic effect in mild cognitive impairment patients.^46^, ^47^ This process may be related to the D‐amino acid oxidase inhibition activity of sodium benzoate.^47^ The glycine conjugation pathway is also related to the therapeutic benefits of benzoate and disposal of waste nitrogen,^44^ with nitrogen playing a role in AD‐related neurotoxicity through air pollution and biological ammonia waste products.^48^, ^49^

In the intercept pathway analysis, the “lymph angiogenesis” pathway from the Pathway Interaction Database (PID) and the “*PRC2* methylates histones and DNA” pathway from the Reactome database were notably associated with at least one endophenotype within each A/T/N/C category. The lymph angiogenesis pathway relates to *VEGFR3* signaling in the lymphatic endothelium,^50^ which plays a role in immune function through vascular permeability.^51^ The *VEGF* family of genes has been implicated with cognitive decline in AD.^52^, ^53^ The *PRC2* pathway relates to transcriptional regulation of genes through methylation and histone modification,^54^ and *PRC2* has been shown to regulate the expression of AD‐related genes such as *Wt1*, *APP*, and *PSEN1*.^55^ The *RAS/MAPK/ERK* signaling cascade pathways showed strong patterns of association across different clusters of endophenotypes and play a significant role in a number of signal cascades, largely related to cell proliferation, differentiation, and survival.^56^, ^57^ As such, these pathways play a role in a large number of diseases, including AD.^58^

In the SNP×Time longitudinal trajectory pathway analysis, a few notable patterns of association are present with cross‐endophenotype association. The “*PI3K* events in *ERBB4* signaling” has a robust association with neurodegeneration endophenotypes (MRI and FDG). Neuregulin signaling through *ERBB4* and mediated by *PI3K* may play an important role in neuronal Aβ toxicity.^59^ Downstream *PI3K*/*AKT* signaling may play a role in other AD‐related processes including tau phosphorylation, insulin signaling, and autophagy.^60^ The “receptor‐type tyrosine‐protein phosphatase” pathway, associated with EF and frontal lobe cortical thickness, plays a role in synaptic organization and is involved with *IL1RAP*,^61^ which has been implicated in AD.^62^, ^63^ *RhoA*/*ROCK* signaling, associated with cognition and medial temporal thickness, has been implicated in promoting AD as a feedback loop with Aβ production and neurofibrillary tangles.^64^ Glycosylation shows a robust association across MRI, cognitive, and phosphorylated tau endophenotypes. Aberrant protein glycosylation has been implicated in AD, including the formation of Aβ plaques.^65^ *APP* and proteins involved in its cleavage pathway have been shown to be altered at N‐glycosylated sites. N‐glycosylation also influences neurofibrillary tangle formation.^66^ The N‐glycosylation pathway is implicated in this study, specifically the N‐glycan precursor biosynthesis ALG7 to ALG11 pathway. Fucosylation is the addition of fucose in an N‐glycan, O‐glycan, or glycolipid,^67^ a process that relies on GDP‐fucose synthesis that is implicated in this study. A preliminary study has shown that L‐fucose is depleted in mouse 5xFAD and human brains.^68^ Fucosylation through *FUT8*, downstream and reliant on the presence of GDP‐fucose, has been shown to be increased in microglia in human AD and 5xFAD mice, impacting inflammatory pathways.^69^

Several limiting factors influence longitudinal studies. Sample size is a limitation in GWASs and is compounded when working with longitudinal data. Longitudinal observation has challenges in acquiring every biomarker at every visit, creating missing time points for many participants. The LMM approach is well suited for handling this form of variable availability and incorporating it into the error term, but it still might introduce limitations on the ability to fully model disease trajectory. Data quality and visit‐to‐visit variability pose much greater risks in longitudinal analysis. Efforts have been made to best account for this variability through covariates and selection of endophenotypes with lower visit‐to‐visit variability, but variability still contributes as a possible error source. This study is also limited by the participant population. While this helps reduce population stratification, the non‐representative sample may limit the generalizability of the findings to other populations. However, large‐scale efforts are under way to address AD genetics in multiethnic populations. ADNI's focus on increasing racial and ethnic diversity in its latest phases will help overcome this limitation in the future. Tau‐PET data are available in ADNI‐3, but at the time of this study, it was significantly lower in sample size compared to other measures and insufficient for genetic analysis. A meta cross‐sectional study has been performed separately to address tau‐PET,^70^ and as more data become available in ADNI and other datasets, this method will be considered for future longitudinal analyses.

Age at observation was used as the time variable in this analysis, representing the disease over the course of a lifetime as possible within the dataset. Alternative models of aligning the data may prove useful in identifying genetic effects that contribute to changes in longitudinal trajectory. Age of onset varies by individual and is not well accounted for due to the lack of precise onset data for many participants. Future studies will look to incorporate unbiased methods for aligning the data to provide more power in identifying a change in trajectory in relation to AD presentation. Other methods could be used to break down longitudinal trajectories into subgroups, which may provide further insight into heterogeneity in AD.

This study identified many pathways from associations with biologically supported genetic effects. Many of these pathways are associated with biological pathways that have been implicated in AD. The findings here provide new insights into specific perturbations in genetic pathways and steps within those pathways that can be targeted for therapeutic approaches. Further study, such as through cell models or animal models, is needed to determine more precise functional effects relating to these pathways. Utilizing longitudinal endophenotypes for genetic analysis provides a means to break down AD heterogeneity and understand genetic effects that contribute to individual variation in disease presentation and progression. Fully understanding the genetic effects and pathways that contribute to AD risk and AD pathological trajectory is crucial to developing the personalized medicine approaches that will be necessary in AD treatment strategies.

## CONFLICT OF INTEREST STATEMENT

Dr. Saykin receives support from multiple NIH grants (P30 AG010133, P30 AG072976, R01 AG019771, R01 AG057739, U19 AG024904, R01 LM013463, R01 AG068193, T32 AG071444, U01 AG068057, U01 AG072177, and U19 AG074879). He has also received support from Avid Radiopharmaceuticals, a subsidiary of Eli Lilly (in kind contribution of PET tracer precursor) and has participated in scientific advisory boards (Bayer Oncology, Eisai, Novo Nordisk, and Siemens Medical Solutions USA, Inc.) and an Observational Study Monitoring Board (MESA, NIH NHLBI), as well as External Advisory Committees for multiple NIA grants. He also serves as editor‐in‐chief of *Brain Imaging and Behavior*, a Springer‐Nature journal. Dr. Li Shen has served as a consultant on NIH Grant R24 EB029173 from the University of Massachusetts. Dr. Tatiana Foroud acts as a consultant for NIH‐funded centers and other infrastructure grants and receives funding for travel expenses from the Michael J. Fox Foundation and from academic institutions at which she serves as an advisor. Dr. Sujuan Gao has served as an unpaid board and committee member on the data safety monitoring board for R01 AG058586, 18G‐MC‐LMDC, and R01 HL151951 and paid data safety monitoring board member for R01 AG061898. Dr. Shannon Risacher served as Communications Chair, unpaid, for the Alzheimer's Association AWARE PIA and has received travel funding for the Charleston Conference on Alzheimer's Disease. She also has an equity interest in Eli Lilly (<$10,000), a company that might benefit from the research results of this study. Dr. Thea Rosewood, Dr. Shiwei Liu, and Dr. Kwangsik Nho have no interests to declare. The funders had no role in the design of the study; in the collection, analyses, or interpretation of data; in the writing of the manuscript; or in the decision to publish the results. Author disclosures are available in the Supporting information.

## Supporting information



Supporting Information
